# Fatigue and recovery-related changes in postural and core stability in sedentary employees: a study protocol

**DOI:** 10.3389/fphys.2024.1490041

**Published:** 2024-12-24

**Authors:** Banafsheh Amiri, Erika Zemková

**Affiliations:** Department of Biological and Medical Sciences, Faculty of Physical Education and Sport, Comenius University in Bratislava, Bratislava, Slovakia

**Keywords:** Abt’s trunk muscle fatigue protocol, electromyography, foam rolling exercises, inertial sensor system, posturography, sedentary adults, trunk stabilization exercises

## Abstract

Prolonged sitting leads to a slumped posture, which indirectly influences spinal curvature and increases low back and hamstring stiffness. Active rather than passive recovery is an effective way to reduce the risks associated with such prolonged inactivity. However, it remains to be investigated which of the exercises frequently used for this purpose, the trunk stability and foam rolling exercise, is more beneficial. This protocol study will compare the effects of foam rolling exercises on the recovery of impaired core and postural stability induced by core muscle fatigue and hamstring muscle stiffness with those of trunk stabilization exercises in sedentary adults. Twenty sedentary adults ranging in age from 25 to 44 years old, comprising 50% men and 50% women, will participate in a modified Abt’s trunk muscle fatigue protocol, then proceed with (1) active recovery in the form of trunk stabilization exercises, (2) active recovery in the form of foam rolling exercises, and (3) passive recovery, entails lying on a bed, respectively. Pre-fatigue, post-fatigue, and after all three recovery modalities, core and postural stability, and back and hamstring muscle flexibility will be evaluated using an inertial sensor system, and a posturography system. Muscle-fatigue conditions will be determined using electromyogram signals. Although the effects of foam rolling and trunk stabilization exercises can be attributed to different physiological mechanisms, the former releasing myofascial to improve flexibility and reduce muscle tension, the latter strengthening core muscles to stabilize posture, we hypothesize that both are equivalently effective in reducing the consequences of prolonged sitting.

## 1 Introduction

The modern lifestyle has led human societies toward a more sedentary existence (Stockwell et al., 2021; Chaudhary et al., 2024). The literature indicates that, in the future, three-fourths of all jobs worldwide will be sedentary (Khanzadeh et al., 2020). Sedentary behavior is described as “any waking activity with an energy expenditure of 1.5 METs or less while sitting, reclining, or lying down.” (Lassalle et al., 2021). Given that desk workers spend a considerable portion of their time seated, they could be potential targets for interventions aimed at reducing sedentary behavior (Barone Gibbs et al., 2018). This type of lifestyle is associated with musculoskeletal disorders, particularly when sitting is prolonged in uninterrupted bouts (Chia et al., 2015).

Prolonged sitting with ongoing contraction of the trunk muscles can lead to fatigue in the deep trunk muscles (Waongenngarm et al., 2016; Waongenngarm et al., 2018; Saiklang et al., 2022a; Saiklang et al., 2022b), which, in turn, can alter the neuromuscular system and result in core stability disorders (Jabłońska et al., 2021). This is because the neuromuscular system is responsible for creating the conditions necessary for adequate core stability through the coordinated contraction of the trunk muscles (Esmaeili and Askari, 2020).

Also, muscle fatigue can affect postural stability. Under fatigue, afferent feedback is disrupted, impairing joint proprioception and subsequently affecting somatosensory input (Tajali et al., 2022). Reduced efficiency in muscle contraction (Tajali et al., 2022), inadequate processing of sensory input, and compromised neuromuscular performance due to fatigue can disrupt both feedforward and feedback control of postural sway (Zemková et al., 2021a). Typically, a decline in postural stability is observed with muscular fatigue (Akulwar and Mulgaonkar, 2017). Especially, strategies of postural control can alter under muscle fatigue (Johanson et al., 2011). For instance, a study by Johanson et al. showed that the strategies used by fatigued individuals, when faced with increased postural demands, are similar to those utilized by individuals experiencing recurring low back pain. Fatigued healthy individuals exposed to a sudden perturbation have demonstrated an elevation in electromyographic amplitude, longer activation latencies, diminished muscle activity, and heightened co-contraction (Zemková et al., 2021a; Zemková et al., 2021b).

Another potential risk factor for impaired postural stability induced by prolonged sitting is the shortening of hamstring muscles due to repetitive keeping knees in flex (Shamsi et al., 2020). The hamstring’s structure includes a biceps femoris torso linked to the ischial tuberosity, an extension of the Sacro tuberous ligament, which crosses the os sacrum and attaches to the thoracolumbar fascia (Na’ima et al., 2019). Through this relationship, an increase in posterior pelvic tilt and reduction in lumbar lordosis due to the shortening of hamstring muscles can result in a flat back (Fatima et al., 2017; Na’ima et al., 2019; Encarnación-Martínez et al., 2023). Changes in the lumbar curvature can shift the center of gravity, potentially impairing postural stability (Shamsi et al., 2020; Encarnación-Martínez et al., 2023).

Given the fact that impaired core and postural stability strongly correlates with hamstring and trunk muscle corset conditions, it becomes crucial to restore optimal muscle capacity and efficiency. Physical exercises offer a potentially feasible option to reduce sedentariness at work without major disruption to office work practices. During core exercises, fatigued trunk muscles show an increase in EMG root mean square amplitude values. Specifically, two studies demonstrated that the abdominal drawing-in maneuver (Saiklang et al., 2021), as well as a combination of the abdominal drawing-in maneuver with supported dynamic lumbar extension (Saiklang et al., 2022a), increased the activation of the transversus abdominis (TrA) and internal oblique (IO) muscles. Additionally, the abdominal drawing-in technique led to higher activation ratios of TrA and IO compared to the rectus abdominis, highlighting its effectiveness in engaging these key muscles for spinal support. In another study, Abdelraouf and Abdel-Aziem demonstrated that core stability interventions can alter the recruitment patterns of core muscles (Abdelraouf and Abdel-Aziem, 2016), and effectively improve the fatigue resistance of these muscles, thereby restoring core stability (Zou et al., 2021). Trunk stabilization exercises aim to enhance and regain the coordinated contraction of both local and global muscles, improving control over the pelvis and lumbar spine (Richardson et al., 1999; Hodges, 2003). This also helps the trunk muscles regain their ability to fulfill the requirements of postural control (D’souza et al., 2022). Training with trunk stabilization exercises has shown significant improvements in core stability (Butcher et al., 2007; Sato and Mokha, 2009), and immediate improvements in static and dynamic balance (Kaji et al., 2010; Imai et al., 2011; Imai et al., 2014a; D’souza et al., 2022). Also, Szafraniec et al. demonstrated improvement in the mediolateral body balance after core exercise (Szafraniec et al., 2018). These effects were noticeable within a span of 30 min post-exercise and persisted for a minimum of 24 h. An enhanced automatic response in maintaining a stable upright posture was observed 24 h after the exercise session. However, there has been little discussion so far about the efficiency of trunk stability exercise in the recovery of core and postural stability during prolonged sitting at the workplace.

In recent times, foam rolling exercises have become increasingly popular due to their affordability, ease of use, and time-saving benefits (Kipnis, 2016; Biscardi et al., 2021; Zahiri et al., 2022). This method involves self-massage, where a person applies body weight on the foam roller to roll and compress specific muscle groups (Healey et al., 2014; Zahiri et al., 2022; Huang et al., 2024). Thus, instead of a trainer providing guidance, foam rolling can be carried out independently by an individual (Yektaei et al., 2023; Kalichman and David, 2017). These exercises help regulate muscle imbalances (Costa et al., 2017; Madoni et al., 2018; Egesoy et al., 2020; Hamzeh Shalamzari et al., 2020; Acar et al., 2022), alleviating muscle pain (Beardsley and Škarabot, 2015; Wiewelhove et al., 2019; Egesoy et al., 2020; Hendricks et al., 2020; Cabrera-Martos et al., 2022; Pagaduan et al., 2022) and joint stiffness (De Benito et al., 2019; Egesoy et al., 2020; Yokochi et al., 2024), and enhancing neuromuscular tone, flexibility (De Benito et al., 2019; Egesoy et al., 2020), and activity in the musculotendinous complex, promoting the maintenance of normal functional muscle length (Healey et al., 2014; Ateş et al., 2018; Egesoy et al., 2020; Nakamura et al., 2021). Using these exercises can lead to an expansion of range of motion and a reduction in muscle stiffness, without compromising muscle strength and athletic performance (Nakamura et al., 2021). The benefits of these exercises are attributable to changes in a muscle’s viscoelastic properties, decreasing fascial tenderness through the activation of Golgi tendon organs and mechanoreceptors, and possibly an increase in angiogenesis and vascular endothelial growth factor (Kurt et al., 2023; Behm and Wilke, 2019; Naderi et al., 2020). There are also psychological benefits, such as reducing anxiety and enhancing mood and relaxation (Kurt et al., 2023; Kerautret et al., 2021). 4-Week foam rolling exercises can be applied to improve the back and hamstring muscles’ range of motion (Sherer, 2013; Junker and Stöggl, 2015). Additionally, three 20-min sessions of foam rolling exercise following an exercise-induced muscle damage protocol, administered at 0, 24, and 48 h, improved passive and dynamic range of motion (MacDonald, 2013). The foam roller, due to its unstable surface, challenges the body to maintain stability and balance during exercise (Lukas, 2012; Junker and Stöggl, 2019). Furthermore, it appears that foam rolling exercises may contribute to restoring core and postural stability by reducing hamstring muscle stiffness and relaxing trunk muscles. However, only a limited number of studies have examined the effect of foam rolling exercises on core and postural stability parameters (Grabow et al., 2017; Junker and Stöggl, 2019; Alim, 2021).

As explained earlier, the mechanisms for the effects of trunk stabilization exercises and foam rolling exercises on core and postural stability are described differently. It is hypothesized that both exercises will induce a significant improvement in core and postural stability. If foam rolling is found to be an effective exercise for recovery of impaired core and postural stability, core-specific exercises may not be necessary in the workplace. This would lead to a reduction in training time (Zahiri et al., 2022), which is a positive aspect of workplace exercise. This study presents a protocol for the investigating the modeling of the process of muscle fatigue and recovery to provide an opportunity to identify suitable workplace exercises. The purpose of this study will be to compare the immediate effect of trunk stabilization exercises on recovery of impaired core and postural stability induced by fatigue with that of foam rolling exercises in sedentary adults.

## 2 Materials and methods

### 2.1 Study design

**FIGURE 1:**
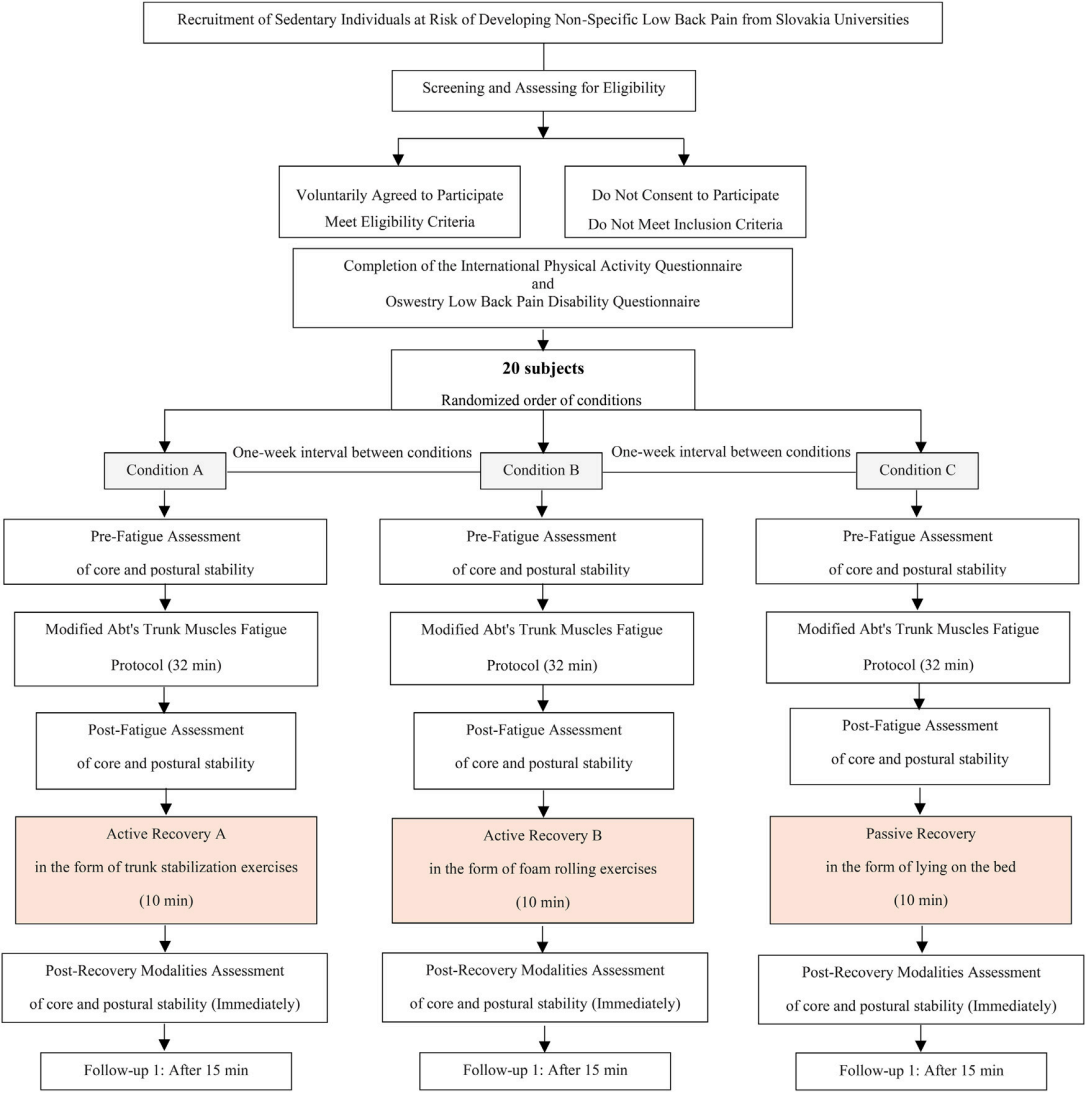
Flow diagram of the study protocol.

The purpose of this pre- and post-quasi-experimental study will be to compare the immediate effect of trunk stabilization exercises on recovery of impaired core and postural stability induced by fatigue with that of foam rolling exercises in male and female sedentary adults. The protocol will be implemented and documented according to the Standard Protocol Items: Recommendations for Interventional Trials Statement (SPIRIT). The study design is provided in Figure 1. The research has obtained approval from both the ethics committee at the Faculty of Physical Education and Sport, Comenius University in Bratislava (Approval No. 5/2022), as well as the Ethics Committee of Kerman University of Medical Sciences (Approval No. IR. KMU.REC.1401.386). The study was also registered in the Clinical Trials system under the reference IRCT20221126056606N1.

#### 2.1.1 Ethical considerations and informed consent

This study will be conducted by the ethical principles outlined in the Declaration of Helsinki. All participants will be provided with detailed information about the purpose, procedures, potential risks, and benefits of the study. They will be informed that their participation is voluntary, and they will have the right to withdraw from the study at any point without any consequences. Written informed consent will be obtained from each participant before their inclusion in the study.

#### 2.1.2 Data and safety monitoring plan (DSMP)

This study involves non-invasive interventions, so a formal Data Safety Monitoring Plan (DSMP) or Data Safety Monitoring Board (DSMB) will not be implemented. However, the research team will actively monitor participant safety throughout the study and address any adverse events promptly. All procedures are designed to minimize risk and ensure participants’ wellbeing.

### 2.2 Participants and setting

A total of 20 sedentary employees of universities ages 25–44 years, comprising 50% men and 50% women, will participate in this study. This study will be carried out at the Faculty of Physical Education and Sports, Comenius University in Bratislava, Slovakia.

### 2.3 Inclusion and exclusion criteria

Participants will be selected based on the following criteria: Sedentary adults aged 25–44 years who report sitting continuously for a minimum of 2 hours on any given working day (Saiklang et al., 2022a). The International Physical Activity Questionnaire [IPAQ] will be used to screen potential study participants to determine whether they are sedentary. Potential participants who meet any of the following criteria will be excluded: a history of femoral, spinal, or intra-abdominal surgery within the past 12 months (Sitthipornvorakul et al., 2015); pregnancy (Abboud et al., 2014); participants having been diagnosed with congenital arthritis, spine and disc infections, rheumatoid arthritis, spondylolisthesis, ankylosing spondylitis, spinal anomalies, spondylosis, tumors, systemic lupus erythematosus, or osteoporosis will be excluded (Sitthipornvorakul et al., 2015). Additionally, individuals who have received physiotherapy services within 1 month prior to the study’s commencement (Fortun-Rabadan et al., 2021) or have experienced any form of mental health disorder, including depression and anxiety, within the last 3 years (Fortun-Rabadan et al., 2021) will also be excluded. Furthermore, participants will undergo health screenings before the study to rule out infections or symptoms of fatigue (White, 1997). They will also disclose any musculoskeletal disorders experienced in the last 6 months, including acute or chronic low back pain. To ensure the validity of the inclusion and exclusion criteria regarding low back pain, we will administer the Oswestry Low Back Pain Disability Questionnaire (Fairbank JC 1980) for pain assessment. Additionally, a BMI <30 will be used to screen for overweight or obesity. To further ensure only metabolically healthy individuals are included, participants with major medical disorders or metabolic conditions such as diabetes, hypertension, hyperlipidemia, or related diseases will be excluded.

#### 2.3.1 International physical activity questionnaire

The International Physical Activity Questionnaire [IPAQ] will be used to identify those who are sedentary in order to screen potential study participants The IPAQ is a standardized self-report instrument specifically crafted to evaluate physical activity levels within an individual’s typical daily routine (Mehta et al., 2018). There are two versions of this questionnaire, long and short (Demircioğlu et al., 2021). The IPAQ Long Form (IPAQ-LF) is typically employed in clinical settings or research studies to gauge physical activity levels. In contrast, the IPAQ Short Form (IPAQ-SF) is utilized to screen physical activity levels within the general population (Mehta et al., 2018). The objective of both versions of the questionnaire is to evaluate the quantity and intensity of physical activity that an individual engages in on a weekly basis (Mehta et al., 2018). Each version of the questionnaire encompasses four domains of physical activity, which include work-related activities, leisure-time physical activity, domestic and gardening activities, and transport-related activities. Additionally, sedentary time, defined as time spent in a seated or reclined position during waking hours, is recorded for both a typical weekday and weekend day within the same time frame (Ruescas-Nicolau et al., 2021). In the present study, the long version IPAQ will be used, which contains 27 items across four domains (Mehta et al., 2018). With the long form, it is possible to compute scores specific to each domain, as well as scores tailored to individual activities, and continuous scores. Continuous scores are expressed in metabolic equivalent minutes (MET) as a metric for quantifying physical activity. People are categorized into low, moderate, or high levels of physical activity based on these scores (Mehta et al., 2018).

### 2.4 Sample size estimation

Sample size estimation was conducted utilizing the G*Power software package (version 3.1.9.7) based on the methodology outlined in the study by Fonta et al. (2021). The input parameters included the following: the statistical test was repeated measures, within factors; the α error probability was 0.05; the effect size F was 0.25; power (1 − β error probability) was 0.80; correlation among repeated measures was 0.70; and no sphericity correction (e = 1) was applied. It is important to note that the magnitude of the correlations (r = 0.7) can be considered large according to Hopkins et al. (2009). This correlation was chosen to reflect the anticipated relationship between the repeated measures in this study, based on the literature (Hopkins et al., 2009; Fonta et al., 2021; Zahiri et al., 2022). Consequently, the total sample size was determined to consist of seven subjects (in each trial). To ensure ample statistical power, especially in scenarios with smaller-than-expected effects or lower correlations between repeated measures, we decided to include 20 participants, who will be tested in the three experimental conditions. This approach also takes into account the possibility of participant dropouts during the study.

### 2.5 Procedures

The study will follow a structured protocol consisting of two main phases: familiarization and experimentation. During the familiarization phase, participants will be introduced to the fatigue protocol, recovery modalities, and assessment tools. In the experimentation phase, the modified Abt’s fatigue protocol will be implemented, where participants perform core exercises aimed at inducing fatigue in the trunk muscles. This will be followed by different recovery modalities. The evaluation will include pre-fatigue, post-fatigue, and post-recovery assessments, focusing on muscle fatigue, postural and core stability, and the flexibility of the back and hamstring muscles.

#### 2.5.1 Familiarization

In the first phase, a familiarization session will be performed about 1–2 h before data collection. Participants will be informed about the study’s objectives and procedures prior to participating in the research. During the second phase, participants will be tasked with completing a questionnaire detailing their baseline and personal characteristics. Height will be assessed using a single stadiometer, while body mass will be measured with a calibrated digital scale. The weights utilized in the fatigue protocol will be determined individually for each participant through the familiarization process. This involves finding the heaviest weight that participants can perform correctly 20 times in 40 s (Askari and Esmaeili, 2021). Throughout this period, participants will be instructed to maintain their regular diet and avoid engaging in strenuous exercise or consuming performance-enhancing energy drinks, medications (such as antidepressants or pain medications), drugs, or caffeine for 48 h leading up to the data collection sessions (Williams et al., 1987; Tarnopolsky, 2008; Armstrong et al., 2018; Bergefurt, 2023).

#### 2.5.2 Experimentation

The experiment involves 20 participants, each completing three sessions spaced 1 week apart. In each session, they will perform a modified Abt’s fatigue protocol, consisting of eight exercises targeting their core muscles. Subsequently, they undergo recovery modalities. Everything will remain the same throughout the sessions except the type of recovery modality, which will be varied (trunk stabilization exercises, foam rolling exercises, and passive recovery). The order of the recovery modalities will be counterbalanced to control for potential sequence effects. Data will be gathered from participants at three different time points:

1. Pre-fatigue assessment (baseline measurement).
2. Post-fatigue assessment (immediately following the fatigue protocol).
3. Post-recovery modalities assessment (immediately and 15 min after the completion of active and passive modalities, respectively).

Participants will be tested by the same examiners at the same time.

#### 2.5.3 Data collection

##### 2.5.3.1 Primary outcomes

###### 2.5.3.1.1 Assessment of the trunk and hamstring muscle fatigue

Electromyography signals from the hamstring and trunk muscles will be recorded using the Delsys Trigno™ wireless EMG system. These muscles will be the erector spinae muscles at level L1 (Elfving et al., 2002a), the lumbar multifidus at the L5 level (Larivière et al., 2003), and medial hamstring muscles (Fiebert et al., 1997). These measurements will be taken at baseline, post-fatigue protocol, and post-recovery modalities (immediately and after 15 min). Following skin abrasion and cleansing with alcohol, electrode pairs will be placed bilaterally on the targeted muscles (Elfving et al., 2002b). The inter-electrode distance will be 2.5 cm (Areeudomwong et al., 2012a) on erector spinae muscles and the lumbar multifidus. The electrode for the medial hamstring will be positioned midway between the ischial tuberosity and the medial epicondyle of the tibia. During the tests for maximum isometric strength of the back extensor and hamstring muscles, sEMG recordings will respectively be collected from the multifidus and longissimus muscles (erector spinae), as well as from the medial hamstring muscles. Each sEMG measurement episode will have a standardized duration of 5 s, during which continuous recording occurs. The raw sEMG signals will be recorded with a sampling frequency of 2000 Hz and the bandwidth of sEMG will be around 20Hz–450 Hz (Larivière et al., 2003). The characteristics of muscle fatigue detected by EMG include a shift from a high-frequency spectrum to a low-frequency spectrum and an increase in amplitude (Sakurai et al., 2010). The assumption is that recovery and fatigue are mutually exclusive. Therefore, the shift from a low-frequency spectrum to a high-frequency spectrum and a decrease in amplitude are indicative features of muscle recovery detected by EMG. In this study, the absolute values of the frequency features (median frequency MDF, and mean power frequency, MPF) and the mean amplitude (root mean square, RMS) of the sEMG signals during the middle 3 s of the 5-s testing period will be employed to assess muscle fatigue and recovery. To ascertain these variables, the raw EMG signal will undergo processing using a fast Fourier transformation.

###### 2.5.3.1.2 Assessment of the postural and core stability

The participants will stand on a force plate without shoes, with their arms naturally hanging by their sides. They will be asked to maintain an upright position with one foot directly in front of and touching the other foot. Participants will self-select the forward foot. Postural stability measurements will be taken at baseline, post-fatigue protocol, and post-recovery modalities (immediately and after 15 min).

Trials will be conducted under a variety of conditions in a randomized order: (1) Tandem stance on a force plate with eyes open, (2) Tandem stance on a force plate with eyes closed, (3) Tandem stance on a foam mat (Airex Balance Pad) positioned on the force plate with eyes open, (4) Tandem stance on a foam mat (Airex Balance Pad) situated on the force plate with eyes closed (Zemková et al., 2016). Each participant will complete one set lasting 30 s under each condition. There will be a short break between every two trials (Zemková et al., 2021a).

####### 2.5.3.1.2.1 Measurement of center of pressure (CoP) variables under stable and unstable conditions

The FiTRO Sway Check (FiTRONiC, Bratislava, Slovakia) will be utilized for conducting an assessment of postural stability. The system detects the real force at the corners of the force plate and computes the instantaneous CoP position. It operates at a sampling rate of 100 Hz, with a 12-bit analog-to-digital signal conversion. The resolution of the CoP position is finer than 0.1mm, and it can measure within a range of 0–1,000 units per second. Non-linearity is within ±0.02% of the full scale, with a combined error of 0.03%. Sensitivity is at 2 mV/V±0.25%, and each sensor has an overload capacity of up to 150% of its range. Repeated measurements showed that reliability of CoP variables is consistently high, ranging from good to excellent, with no noteworthy fluctuations observed across consecutive days. The Romberg quotient, which involves calculating the sway ratio with eyes closed *versus* eyes open (EC/EO), will also be computed. Postural stability variables will be recorded using the FiTRO Sway Check (FiTRONiC, Bratislava, Slovakia) during unstable conditions. The apparatus comprises a square platform upheld by four springs possessing an elasticity coefficient of 40N/mm. When the center of pressure is shifted horizontally, the body weight distribution changes at the four corners of the platform. The force at each corner is determined by multiplying the spring’s elasticity coefficient by the vertical distance measured using a precise sensor. Analog signals are converted to digital (AD-converted) and sampled by computers at a rate of 100 Hz. Calculations of the instantaneous CoP position relies on the distribution of force across the four corners of the platform. Basic parameters of postural stability (i.e., mean CoP displacements in medio-lateral and anterior-posterior directions and mean CoP velocity) will be analyzed (Zemková et al., 2021b).

####### 2.5.3.1.2.2 Measurement of center of mass (CoM) variables under stable and unstable conditions

Concurrently, the Gyko inertial sensor system (Microgate, Bolzano, Italy) will be employed to evaluate center of mass (CoM) variables. These sensors will be fastened with an elastic belt on the participant’s posterior trunk, close to the body’s center of mass. The height of the Gyko device positioned on the trunk will be adjusted prior to measurement to mitigate any potential influence on the collected data. The Gyko system comprises a 3D accelerometer for measuring linear accelerations experienced by the device, a 3D gyroscope for measuring the device’s angular velocities, and a 3D magnetometer for measuring the magnetic field surrounding the device. It provides data measurements at a frequency of up to 1,000 times per second (1 kHz), ensuring a high temporal resolution of the collected data. Using these data as a foundation, specialized software algorithms are employed to delineate the kinematics of the analyzed body segment. It calculates three primary measures of body sway: sway travel speed, sway length and area, and sway frequency (Zemková et al., 2021a).

##### 2.5.3.2 Secondary outcomes

###### 2.5.3.2.1 Assessment of the back and hamstring muscles’ flexibility

The flexibility of right and left trunk side flexion and rotation, and also a range of movement hip flexion will be measured using the Gyko inertial sensor system (Microgate, Bolzano, Italy). This device can supply acceleration (up to 16 g), angular velocity (up to 2000°/s), and magnetic field measurements via a built-in 3D accelerometer, a 3D gyroscope, and a 3D magnetometer, respectively, with an acquisition frequency of 1,000 Hz. Using Bluetooth 4.0, data will be streamed to a computer with dedicated software (Gyko Repower; Microgate, Bolzano, Italy). It will be possible to display and store it for future use and analysis using computer software (Fonta et al., 2021). A good reliability and excellent concurrent validity of this device have been revealed (Hamersma et al., 2020).

####### 2.5.3.2.1.1 Measurement of back muscles flexibility

The device will be attached to a vest, which will be worn and adjusted on each subject’s upper torso during testing. So, the sensor will be located between the two scapulae at the level of the roots of the scapulae spines. The active range of trunk side flexion will be measured with each subject in the upright standing position while facing and maintaining the palmar surface of their hands in contact with the wall. Each subject will be advised to flex their trunk to the right and left sides by sliding their hands on the wall, avoiding forward bending or twisting of the trunk. They will keep their elbows and knees straight, as well as their heels on the ground. The active range of trunk rotation will be measured in the horizontal level with each participant in the upright sitting position. The effects of the potential lower body and pelvic movements on trunk rotation measurements will be prevented by instructing each subject to gently pressure a cylindrical cushion that will be placed between their knees (Fonta et al., 2021).

####### 2.5.3.2.1.2 Measurement of hamstring muscle flexibility

To measuring the range of movement hip flexion a standard procedure for the passive straight leg raise test will be adopted. Subjects will be first instructed to lie supine on a medical bed. The Gyko will be strapped at the level of the distal end of the femur of the tested leg. Inelastic straps will be used to fix the contralateral limb to the medical bed. The tested leg will be then passively lifted in full extension to the limit of the available range of motion, or the point the subjects started to feel pain or discomfort. The procedure will be repeated for both limbs. The testing order of the limbs will be randomized across subjects (Thomas et al., 2022).

Flexibility measurements will be performed at baseline, post-fatigue, and following the recovery modality (immediately and after 15 min).

#### 2.5.4 Fatigue protocol

Fatigue in the trunk muscles will be induced using the modified Abt protocol (Abt et al., 2007). This protocol consists of four sequential cycles, each containing eight exercises, with the entirety of the protocol lasting 32 min. The exercises within each set are organized in the following order: 1) seated trunk rotation using a medicine ball, 2) static torso extension while prone with a medicine ball, 3) supine lower torso rotation with a medicine ball, 4) inclined bench sit-ups with a weight plate, 5) side binding with a weight plate, 6) lumbar extension rotation with a weighted plate, 7) standing trunk rotation with pulley resistance, and 8) holding a supine isometric bridge position. Weight plates will be chosen for each subject on a different day prior to testing. Subjects will use the heaviest weight that allows them to perform each exercise 20 times within 40 s while maintaining proper form. Before initiating the fatigue protocol, a 10-min warm-up will be conducted, comprising 5 min of *in situ* warming followed by 5 min of aerobic stretching, with a focus on the trunk and hamstring muscles. Following the warm-up, the fatigue protocol will commence, during which participants will complete 20 repetitions of each exercise within a 40-s timeframe. There will be a 20-s pause between each exercise. The protocol will end under two conditions: firstly, when participants can no longer maintain correct form during the final set of exercises, and secondly, when they are unable to complete each repetition within a 2-s timeframe during the last set. After each phase of the protocol, the Borg scale will be utilized to evaluate participants’ perceived exertion, ranging from 6 to 20, to monitor fatigue levels (Borg, 1970). A score of six signifies no fatigue. If participants report a score of 17 or higher on the Borg scale at the end of the fourth round, the protocol will end. If they report a lower score, they should continue with another round until they reach a score of 17 (Askari and Esmaeili, 2021).

#### 2.5.5 Recovery modalities

##### 2.5.5.1 Trunk stabilization exercises

The TSE program will consist of (a) Front Plank; (b) Back Bridge; and (c) Quadruped Exercise. Okubo et al. (2010) reported that the trunk stability exercises employed in this study elicit greater activation of local muscles compared to other exercises.

During the front plank exercise, participants will assume a prone position, supporting their body weight with their toes and forearms. From this position, they will lift their right arm and left leg simultaneously, holding them straight for 5 seconds. Then, they will switch to lift their left arm and right leg for another 5 seconds. Afterward, participants will lower their body to the floor and rest for 10 s. This exercise will be repeated five times (Imai et al., 2014a).

During the quadruped exercise, participants will be placed in a four-legged position. They will focus on keeping their pelvis neutral and breathing normally. They will lift their right arm and left leg together, holding them straight for 5 seconds. Then, they will lift the left arm and right leg similarly. After each set, they will rest for 10 s. This exercise will be repeated five times (Imai et al., 2014b).

During the back bridge exercise, participants will lie supine on the floor with their feet flat, knees bent at a 90-degree angle, toes facing forward, and hands folded over their chest. They will raise their pelvis to attain and sustain a neutral hip flexion angle, then elevate one leg off the floor and straighten the knee. This posture will be held for 5 seconds. Subsequently, they will lift the other leg, maintaining the position for 5 seconds. A rest of 10 s will follow. This exercise will be repeated five times (Imai et al., 2014a).

##### 2.5.5.2 Foam rolling exercises

Participants will perform the foam roller exercise targeting the lower extremities and back, focusing on muscles such as the hamstrings, quadriceps, IT band, calves, rhomboids, and latissimus dorsi. They will roll the foam cylinder along each selected area for 30 s, moving from the top of the muscle group to the starting position (Healey et al., 2014).

Quadriceps. For quadriceps, participants lie face down and position the foam roller under their thighs. Their forearms rest on the floor in a plank position. While supporting some of their body weight, participants roll the foam roller distally and proximally from the hip bottom to the knee top (Healey et al., 2014).

Hamstrings and the calves. To target the hamstrings and calves, participants sit with the roller beneath their upper thighs or calves. Their hands are positioned on the floor with fingers facing toward their body. They roll the roller distally and proximally from just below the greater trochanter to just above the knee, or from just below the knee to just above the ankle, while partially supporting their body weight with their hands (Healey et al., 2014).

IT band. For the IT band, participants roll the foam roller while lying on their side. They cross one leg in front of the other and slightly raise the lower leg off the floor. The rolling motion spans from just below the greater trochanter to just above the knee joint (Healey et al., 2014).

Latissimus dorsi. For the latissimus dorsi, participants lie on their side with one arm stretched overhead and the foam roller positioned in the armpit region. Minimal movement is involved in this technique (Healey et al., 2014).

Rhomboids. To target the rhomboids, participants lie on their back with arms crossed and the foam roller positioned beneath their shoulders. They lift their hips off the floor and roll the foam roller downward and upward, from shoulder level to the middle of the back (Healey et al., 2014). The multilevel rigid roller (MRR) will be used in this study (Curran et al., 2008).

##### 2.5.5.3 Passive recovery

Participants will be required to do nothing and lie on a bed in a darkened room for a duration of 24 min between the pretest and posttest assessments (Seidi et al., 2019).

### 2.6 Data management

The study will encompass the collection of individual demographic data and outcome data. The data from the questionnaire will be entered into a database, where the study team will check its accuracy before utilizing it. All information and outcome data will be securely stored on computers with password protection, accessible solely by the study team. Throughout and following the completion of the project, data management will outline the methods for collecting, documenting, storing, and preserving data.

### 2.7 Statistical analysis

The statistical analysis will employ SPSS Statistics software (Version 24; IBM Corporation^©^, United States). To evaluate the normality of core and postural stability, as well as EMG variables, a Shapiro-Wilk test will precede any comparative analyses. For normally distributed data, a two-way ANOVA will be applied to explore the main effects of recovery modalities and time intervals, along with any potential interactions between these factors. The factors under consideration include the type of conditions [trunk stabilization exercises session, foam rolling exercise session, passive recovery] and testing time [T1: baseline, T2: immediately after modified Abt’s fatigue protocol, T3: immediately after recovery modalities, and T4: 15 min after recovery modalities]. To identify significant differences, Bonferroni *post hoc* comparisons will be conducted as necessary.

For non-normally distributed data, nonparametric tests will be utilized. Friedman’s test, followed by Dunn’s *post hoc* test, will be employed to compare core and postural stability, as well as EMG variables across testing times [T1, T2, T3, and T4]. The Kruskal-Walli’s test will be utilized for between-condition mentioned above comparisons at each testing time.

Data will be presented as mean ± standard deviation (SD). Effect sizes for both between-session and within-session comparisons of all quantitative variables will be evaluated using Cohen’s d coefficient: small effect (d < 0.2 and ηp2 = 0.01); moderate effect (d ≈ 0.5 and ηp2 = 0.06); and large effect (d > 0.8 and ηp2 = 0.14) (Cohen, 2013). The significance level (α) will be established at 0.05 for all statistical tests.

## 3 Discussion

Comparing the immediate effects of trunk stabilization exercises to foam rolling exercises provides valuable insights into the efficiency of these two modalities for recovering impaired core and postural stability induced by fatigue in sedentary adults. Prolonged sitting leads to continued contractions of the trunk muscles, resulting in fatigue (Waongenngarm et al., 2016; Waongenngarm et al., 2018; Saiklang et al., 2022a; Saiklang et al., 2022b). Under fatigue, neuromuscular deficits can increase, potentially affecting core and postural control (Jabłońska et al., 2021; Zemková et al., 2021b). Shortness of the hamstring muscle and the posterior pelvic tilt increases while the lumbar spine curvature decreases also during prolonged sitting. These changes can indirectly affects the control of postural and core musculature (Shamsi et al., 2020). Development these abilities can be achieved through regular breaks from prolonged sitting. It is widely accepted that active recovery is superior to passive recovery (Henning et al., 1997; Waongenngarm et al., 2018; Ding et al., 2020), which often includes muscle stretching and strengthening exercises (Macedo et al., 2011; del Pozo-Cruz et al., 2013; Shariat et al., 2018; Sufreshtri and Puspitasari, 2020). However, their specific effects on the recovery of impaired core and postural stability have not been sufficiently investigated so far. Additionally, the question remines as to which of these modalities is more effective and suitable for implementation in workplace conditions.

One of the most frequently used are core stabilization and strengthening exercises. Their goal is to enhance and reinstate the simultaneous contraction and coordination of both global and local muscles (Imai et al., 2014b). Global muscles are connected to the pelvis and hips and are responsible for transferring loads between the thoracic cage and the pelvis, as well as for producing torque (Bergmark, 1989). Local muscles primarily attach directly or indirectly to the lumbar vertebrae (Bergmark, 1989). They play a crucial role in stabilizing the lumbar spine segments, maintaining the lumbar spine in a neutral position, and adjusting functional postures with minimal trunk motions, such as in exercises like the side bridge and back bridge (Imai et al., 2014a). The co-activation, coordination, and neural control of trunk muscles are needed for restoring core and postural stability (Imai et al., 2014b). Evidence supports the efficacy of core stabilization exercises in mitigating muscle fatigue induced by prolonged sitting and static postures (Jackson et al., 2013; Holmes et al., 2015; Kim and Jee, 2020; Saiklang et al., 2021; Salim et al., 2021; Lee et al., 2022; Saiklang et al., 2022a; Saiklang et al., 2022b; Yeom et al., 2023). Trunk stabilization exercises are known to be effective in maintaining core and postural control (Butcher et al., 2007; Durall et al., 2009; Kahle and Gribble, 2009; Imai et al., 2014b). Core exercises, such as McGill stabilization exercises and Brill’s core exercises, have also been found to increase the range of motion actively achieved by the muscles of the back in individuals with chronic low back pain (Cho et al., 2014; Ghorbanpour et al., 2018). This improvement is likely attributed to enhanced coordination among posterior lumbar muscles (Cho et al., 2014; Ghorbanpour et al., 2018).

In the present study, the researcher assumed that foam rolling exercises might improve core and postural stability (Junker and Stöggl, 2019). Foam rolling provides an unstable surface, making it challenging for the body to maintain balance and stability during training. The potential impact on core stability could stem from factors such as the application of one’s own body weight and specific postures during individual exercises, as well as the static effort required during training sessions (Lukas, 2012; Imai et al., 2014a). Though foam rolling exercise are not superior to traditional strengthening exercises on unstable surfaces (Behm et al., 2015), they are sufficient, suitable, and easier to implement for sedentary employees at the workplace.

In addition to improving core and postural stability, these exercises have positive effects on flexibility. The application of foam rolling exercises together with hamstring flexibility improvement (Junker and Stöggl, 2015; Kim and Lee, 2020) could help reduce pathological curves in the spine (Do and Yim, 2018), which, in turn, could lead to improvements in postural stability (Dadfar and Seidi, 2022). Two studies have highlighted the acute effects of foam rolling on muscle flexibility and joint range of motion. Sullivan et al. (2013) showed that a stick roller massage (similar to the principles of foam rolling) leads to an acute increase in hamstring flexibility. Similarly, an acute bout of foam rolling on the quadriceps muscles increased the range of motion of the knee joint (MacDonald et al., 2013). Miller and Rockey (Miller and Rockey, 2006) reported that foam rolling exercises increased hamstring flexibility over an 8-week time period. Although their results indicated gains in range of motion in the intervention group, these values were statistically insignificant when compared to the control group.

When comparing trunk stabilization exercises to foam rolling exercises, a recent study (Zahiri et al., 2022) demonstrated that the traditional prone plank exercise induced lower muscle activation in the dorsal core muscles compared to quadriceps foam rolling. Therefore, individuals seeking time-efficient exercises may still experience back muscle training stress without the need for additional core exercises like planks. While both exercises seem to offer benefits for the recovery of impaired core and postural stability, the fact that foam rolling can be performed independently, without the need for a professional or a second person, adds extra value to this option. Furthermore, it is crucial to recognize that participant motivation and adherence play pivotal roles in achieving successful outcomes with workplace exercises (Kaeding et al., 2017). Performing exercise programs of short duration have been shown (Bell and Burnett, 2009) to improve adherence in employees. Myofascial release is typically recommended to last between 60 and 90 s, up to a maximum of 5 minutes. Various studies support this duration range. One study involved using a roller massager for three sets of 30-s repetitions with 10-s rest intervals, resulting in a 4% increase in ankle range of motion. Another study applied a PVC pipe covered in foam to the quadriceps, conducting two 1-min sessions with 1-min breaks in between, leading to a 12.7% increase in quadriceps range of motion observed 2 minutes after rolling. Additionally, another study examined rolling on the hamstring using different timed intervals: one set for 5 s, one set for 10 s, two sets for 5 s, and two sets for 10 s. This approach yielded a 4.3% increase in range of motion from pre to post-test, with a 2.3% difference observed between 10-s and 5-s intervals (Couture et al., 2015). Employees can take short breaks throughout the day to perform foam rolling routines without disrupting workflow. If foam rolling proves to effectively activate the trunk musculature, it can replace trunk-specific exercises like sit-ups, back extensions, and planks in the workplace (Zahiri et al., 2022). Consequently, training time would reduce (Zahiri et al., 2022), which is a positive aspect for exercise at the workplace. Additionally, research indicates diverse exercise regimens are vital for sustained workplace engagement (Bredahl et al., 2015). Foam rolling’s varied techniques maintain interest and engagement. Employees can perform many different exercises independently, without the need for a coach (Junker and Stöggl, 2019). Furthermore, foam rolling’s massage benefits include relaxation, stress reduction, and improved mindfulness, benefiting mental wellbeing (Wiewelhove et al., 2019). We believe that these exercises, as active breaks, can be successfully implemented to aid in the recovery of impaired core and postural stability in the workplace. These break exercises seem to be capable of restoring muscle strength and flexibility after prolonged sitting, potentially preventing low back pain symptoms in employees.

## 4 Limitations of the study protocol

The results of this study may not directly translate to the general population or the population of young adults with different physical activity levels, because only sedentary young adults will be measured. Furthermore, habitual physical activity of the participants will be assessed only subjectively by IPAQ long version. For example, it has been suggested, that the participants report through IPAQ more moderate-vigorous physical activity and less sedentary time compared with the accelerometer/actigraphy (O’Neill et al., 2017). Furthermore, conducting this protocol in actual workplace settings, where environmental and task-specific factors could naturally induce core muscle fatigue, would provide greater ecological validity. However, the current approach ensures a controlled and standardized environment for inducing and measuring fatigue, enabling robust comparisons of recovery strategies.

## Data Availability

The original contributions presented in the study are included in the article/supplementary material, further inquiries can be directed to the corresponding author.
